# Cracking the Kinase Code: Urinary Biomarkers as Early Alarms for AAA Rupture—A Pilot Study

**DOI:** 10.3390/jcm14113845

**Published:** 2025-05-29

**Authors:** Emma Maria Östling, Tomas Baltrunas, Nathalie Grootenboer, Sigitas Urbonavicius

**Affiliations:** 1 Department of Vascular Surgery, Hospitalsenhed Midt, 8800 Viborg, Denmarksu@biokemi.au.dk (S.U.); 2 Vilnius University Hospital Santaros Klinikos, 08661 Vilnius, Lithuania; 3 Faculty of Medicine, Vilnius University, 01513 Vilnius, Lithuania; 4 Institute of Clinical Medicine, Aarhus University, 8000 Aarhus, Denmark

**Keywords:** abdominal aortic aneurysm, rupture, biomarker, urine, proteome, tyrosine kinase, risk stratification

## Abstract

**Background/Objectives:** Ruptured abdominal aortic aneurysm (RAAA) remains a leading cause of vascular death, with mortality rates approaching 90%. Biomarkers capable of identifying the most at-risk population are urgently needed in the clinic. We aimed to identify potential alterations in the urine proteome that can enable non-invasive detection of abdominal aortic aneurysms (AAA) at high risk of rupture. **Methods**: We used multiplexed kinase inhibitor beads (MIBs) and quantitative mass spectrometry (MIB/MS) to examine potential biomarkers in urine samples. Quantitative proteomic profiling was conducted using iTRAQ labeling and LC-TEMPO MALDI-TOF/TOF analysis, revealing several dysregulated proteins in the urinary proteome between the two groups. MS and MS/MS data were generated using MALDI TOF/TOF instruments (models 5800 or 4800; AB SCIEX). MS/MS spectra were processed with ProteinPilot™ software version 3.0 (AB SCIEX) and matched against the UniProt/Swiss-Prot database for identification of proteins with an Unused ProtScore >1.3. Statistical tests were performed using R/Bioconductor software and bioinformatics analysis using open-source software. **Results**: We quantitatively measured activity over 130 kinases from various kinase families using MIB/MS with a threshold of 1.5-fold change in expression. Statistical analysis assigned significance to EPHB6, AXL, EPHB4, DDR1, EPHA2 and EPHB3. All were tyrosine kinases, and the Ephrin receptor type was dominant. The reduced expression of specific kinases identified by MIB/MS analysis was validated by Western blot. **Conclusions**: This pilot study presents a promising breakthrough in the diagnosis and surveillance of AAA. We identified six dysregulated tyrosine kinases in the urine proteome of patients with RAAAs, suggesting their potential as urinary biomarkers for early detection of AAA at high risk of rupture. However, these preliminary findings require confirmation in larger, prospective cohorts to validate their diagnostic utility and generalizability.

## 1. Introduction

Abdominal aortic aneurysms (AAAs) continue to present a significant clinical challenge due to their often silent progression and the potentially fatal outcome associated with rupture [1]. Improved management strategies are essential to minimizing subsequent aneurysm-related mortality and morbidity. Decision-making regarding intervention in patients with AAA is complex, as both repair and rupture carry high morbidity and mortality. Traditionally, decisions have been based largely on aneurysm diameter [2], a parameter that does not always reliably predict rupture risk. Aneurysms vary in expansion rate and risk of rupture, and these characteristics are not necessarily correlated with diameter [3,4]. Although aneurysms exceeding certain size thresholds are generally scheduled for repair, many of these cases may remain stable, whereas some smaller aneurysms can unexpectedly become unstable. In addition, the available surgical procedures—whether open repair or endovascular repair—carry significant risks of complications, highlighting the critical need for more precise risk stratification of AAA rupture.

In response to this clinical dilemma, recent research has increasingly focused on the identification of novel biomarkers that may provide improved prognostic insights [5]. Although much of the biomarker research to date has concentrated on blood-based markers, emerging evidence suggests that urine, as a non-invasive sample, may offer valuable molecular signatures associated with aneurysm instability [6].

In this context, proteomics—the comprehensive study of the proteome—plays a crucial role. Proteomics enables the detailed mapping of proteins across tissues, cells, and fluids such as plasma [7]. The proteome—representing the full complement of proteins within a cell at a given time—encompasses a diverse array of modified protein isoforms generated through alternative splicing and post-translational modifications [8,9]. As proteins are integral to nearly all cellular functions and regulatory mechanisms, alterations in their expression or structure are closely linked to disease pathology [10]. Accordingly, proteomic analysis provides a valuable approach for both quantitative and qualitative mapping of the proteome, and it may offer critical insights into the molecular mechanisms underlying biological processes in AAA.

Our study leverages proteomic analysis of urine to identify proteins associated with AAA rupture, with the aim of refining current risk assessment models. By integrating proteomic data with existing clinical parameters, we seek to more accurately identify patients at true risk of rupture as a guide in the decision of whether treatment should be surveillance or surgical intervention. This integrative approach not only promises to enhance patient outcomes through more tailored surgical planning but also highlights the potential of urinary biomarkers as a valuable supplement to traditional imaging and blood-based tests in the ongoing effort to predict aneurysm behavior with greater precision.

## 2. Materials and Methods

Urine samples were collected from five patients with RAAA who underwent acute surgery and five patients with non-ruptured AAA who underwent prophylactic surgical treatment. Patient demographics are summarized in Table 1. There were no significant differences between the non-ruptured and ruptured groups in terms of gender, age, aneurysm diameter, body mass index, smoking status, or the use of statins or nonsteroidal anti-inflammatory drugs.

Voided middle portion of urine (100 to 150 mL) was collected immediately before surgery from both RAAA and AAA patients. The urine was kept cold overnight and centrifuged at 2000× *g* for 10 min. The obtained supernatant was then rapidly frozen using liquid nitrogen and preserved at −80 °C for later analysis. Each aliquot contained ∼130 μg of protein, determined by the Bradford assay. Samples of approximately 130 μg were pulverized in liquid nitrogen using a mortar and pestle. The resulting tissue powder was transferred to microcentrifuge tubes and homogenized in a lysis buffer composed of urea (7 M), thiourea (2 M), CHAPS (4% *w*/*v*), and dithiothreitol (100 mM).

We employed a previously established methodology that combines multiplexed kinase inhibitor–conjugated beads (MIBs) with quantitative mass spectrometry (MIB/MS) [11] to investigate potential urinary biomarkers in RAAA and AAA patients. Quantitative proteomic profiling was conducted using iTRAQ labeling and LC-TEMPO MALDI-TOF/TOF analysis, revealing several dysregulated proteins in the urinary proteome between the two groups. The bead-immobilized inhibitors in MIBs compete with ATP for binding at the active site of protein kinases, allowing selective capture of activated kinases for later quantification by mass spectrometry. To visualize the trend in kinase abundance changes between RAAA/AAA samples, we applied a fold-change threshold of ±1.5, based on prior technical replicate analysis and criteria outlined by Unwin et al. [12]. The abundance of active protein kinases in RAAA samples was increased in 42 kinases and reduced in 88, compared to AAA samples. Changes in the urine were assessed using Imagene 9.0 Microarray Quantification and Analysis Software. Mass spectrometry data were processed using the MaxQuant software v. 1.6.12.0 and determined protein 25LFQ (Label-free quantification) intensity, showing the relative amount of protein in the sample compared to the results of the AAA patients.

MIBs were sequentially washed with 20 mL of high-salt buffer and 20 mL of low-salt buffer. Both buffers contained HEPES (50 mM, pH 7.5), Triton X-100 (0.5%), EDTA (1 mM), EGTA (1 mM), sodium fluoride (10 mM), and either 1 M NaCl (high-salt) or 150 mM NaCl (low-salt). Columns were given a final wash with 1 mL of 0.1% SDS before protein elution using 1 mL of 0.5% SDS at 100 °C for 5 min. MIBs were incubated with the prepared urine sample, stirring continuously for 1 h at 4 °C. After incubation, the solution with sorbent was centrifuged at 5000× *g* for 30 s. After discarding the supernatant, the sorbent was washed three times with 500 μL of TBS solution. Proteins were digested using modified trypsin (Promega, Singapore), and the resulting peptides were labeled with iTRAQ reagents (AB SCIEX, Framingham, MA, USA) under light-protected conditions for 2 h, according to the manufacturer’s protocol. Labeled peptides were then purified using PepClean C18 spin columns (Thermo Scientific, Waltham, MA, USA) and fractionated on Tempo™ LC MALDI Spotting System (AB SCIEX) equipped with a Chromolith^®^ CapRod^®^ RP-18e HR analytical column (Merck KGaA, Darmstadt, Germany).

MS and MS/MS data were generated using MALDI TOF/TOF instruments (models 5800 or 4800; AB SCIEX). The MS/MS spectra were processed with ProteinPilot™ software version 3.0 (AB SCIEX) (Paragon algorithm 3.0.0.0, 113442alg3000) and matched against the UniProt/Swiss-Prot database for identification of proteins. Identifications with an Unused ProtScore greater than 1.3 were accepted, corresponding to a confidence level of 95%.

Statistical analyses were performed using R (version 3.6.1)/Bioconductor statistical software (version 3.10) [13,14]. Results were confirmed using STATA (version 16.0) and SAS (version 9.4) statistical software tools. Gene ontology, pathway, and disease analyses were performed using standard databases.

Bioinformatics analysis was performed using open-source software AmiGO (version 2.4.26) [15,16,17], and GeneMANIA (http://www.genemania.org, accessed on 26 May 2025) [18].

A noninterfering assay was used to measure protein concentrations in selected samples (NI Protein Assay, Geno Technology Inc., St. Louis, MO, USA). Equal amounts of protein (5 or 10 µg) from each sample were loaded onto 10–20% or 4–20% Tris-Glycine gels (Invitrogen, Carlsbad, CA, USA) for one-dimensional Western blotting. Following electrophoresis, the separated proteins were transferred onto nitrocellulose membranes. Immunodetection of proteins was performed by incubating the nitrocellulose sheets for a half day at 4 °C in phosphate-buffered saline (PBS), consisting of KCl (2.7 mM), KH_2_PO_4_ (1.8 mM), Na_2_HPO_4_ (10.1 mM), NaCl, (140 mM; pH 7.3), with 0.05% Tween-20 and 5% skimmed milk. The membranes were rinsed in PBS containing 0.05% Tween-20 to eliminate excess blocking agents. Rabbit polyclonal antibodies against EPHB6 0.05 mg/mL (Abcam, Cambridge, UK) and AXL/UFO 0.5 mg/mL (Thermo Fisher, Waltham, MA, USA) were diluted to 1:50–1:200 and 1:2500–1:5000, respectively. The blots were incubated with primary antibodies for 1 h at room temperature, followed by five washes with PBS containing 0.05% Tween-20. Subsequently, membranes were incubated for 1 h with a 1:1000 dilution of peroxidase-conjugated swine anti-rabbit IgG secondary antibody (Dako, Glostrup, Denmark). After a final series of five washes in PBS-Tween, detection was performed using enhanced chemiluminescence (Amersham Biosciences Inc., Piscataway, NJ, USA). Band intensities were quantified with Imaging Densitometer GS710 and Quantity One software (v4.6.9) (Bio-Rad, Basel, Switzerland).

## 3. Results

**Figure 1:**
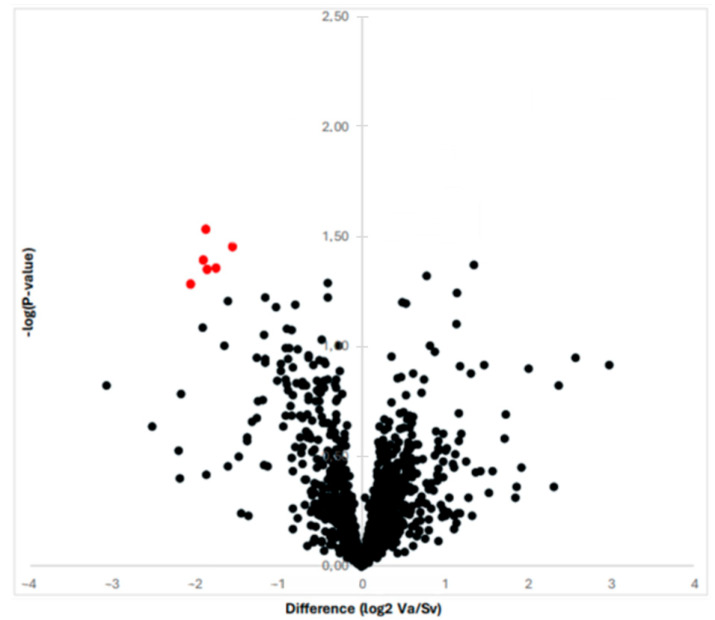
The graph shows the proteins revealed by proteomic analysis with a Vulcano plot. The six most differentially expressed proteins are shown with red color; they all are down-regulated once to the left to the Y-axis.

By means of proteomic analysis, we were able to obtain and compare the protein profiles of the RAAA and AAA specimens. The proteomic analysis is illustrated in Figure 1 with a volcano plot. In total, the MS and MS/MS analysis identified 613 proteins over the 10 specimens. The multiplexed kinase inhibitor-conjugated beads detected a total of 130 protein kinases with altered expression (>1.5 fold). The statistical analysis revealed 13 protein kinases with decreased expression in the RAAA compared to AAA specimens.

A total of six protein kinases were downregulated with statistical significance (*p* < 0.05). All were tyrosine kinases, and the majority were Ephrin receptor type. The identified protein kinases were Ephrin type-B receptor 6 (EPHB6), Tyrosine-protein kinase receptor UFO (AXL), Ephrin type-B receptor 4 (EPHB4), Epithelial discoidin domain-containing receptor 1 (DDR1), Ephrin type-A receptor 2 (EPHA2), Ephrin type-B receptor 3 (EPHB3) (Table 2).

The other seven protein kinases only reached near-significance (*p* < 0.10) due to high within-group variability and are shown in Table 3. These were Nucleoside diphosphate kinase A (NME1), Dual specificity mitogen-activated protein kinase 1 (MAP2K1), Casein kinase I isoform alpha (CSNK1A1), Tyrosine-protein kinase Lyn (LYN), Integrin-linked protein kinase (ILK) Serine/threonine-protein kinase MRCK beta (CDC42BPB) and STE20-like serine/threonine-protein kinase (SLK).

**Figure 2:**
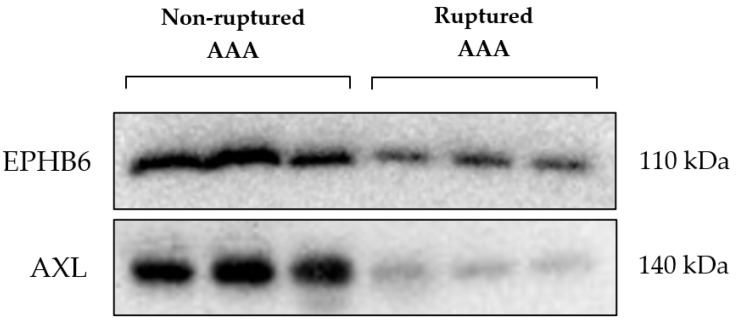
Ephrin type-B receptor 6 (EPHB6); Tyrosine-protein kinase receptor UFO (AXL); Abdominal aortic aneurysm (AAA).

The difference in expression of the kinases EPHB6 and AXL identified by MIB/MS analysis was validated by Western blotting. These kinases were reduced in expression in the RAAA compared to AAA samples, as shown in Figure 2.

## 4. Discussion

The rupture of abdominal aortic aneurysms represents a critical and life-threatening event, demanding a comprehensive understanding of the underlying molecular mechanisms to facilitate the development of effective therapeutic strategies. While the precise etiology of AAA rupture remains multifaceted, involving complex interactions between biomechanical forces and intrinsic biological processes, the dysregulation of intracellular signaling pathways has emerged as a significant area of investigation.

In this study, we have identified six significantly downregulated proteins; all of them were tyrosine kinases and dominated by the ephrin receptor type. It was apparent that some of them could be associated with the formation and development of abdominal aortic aneurysm.

AAA rupture is associated with several molecular and cellular processes, including inflammation, extracellular matrix degradation, and vascular smooth muscle cell (VSMC) dysfunction [19,20,21,22]. Tyrosine kinases are known to be involved in various cellular signaling cascades, including those related to AAA rupture. Several studies have highlighted the involvement of different tyrosine kinase families in vascular pathologies [23,24,25]. The dysregulation of cellular signaling networks, including those involving tyrosine kinases, contributes to the pathogenesis and progression of AAA. Research has shown that vascular smooth muscle cells (VSMCs) derived from patients with AAA exhibit altered tyrosine kinase activity that is crucial for the structural integrity of the aortic wall [26]. VSMC dysfunction plays a role in the weakening of the aortic wall and subsequent risk of rupture [27].

Rombouts et al. identified that the kinase activity of FYN (tyrosine-protein kinase Fyn) was altered in VSMCs derived from patients with AAA compared to healthy controls [26]. This suggests that dysregulation of FYN and potentially other tyrosine kinases may contribute to the pathological changes seen in AAA, such as extracellular matrix remodeling, VSMC dysfunction, and inflammation.

Additionally, the inhibition of Bruton’s tyrosine kinase (BTK) has been shown to ameliorate vascular degeneration, dissection, and rupture in models of aortic aneurysm and dissection (AAD). BTK inhibition reduced macrophage infiltration and modulated macrophage polarization, thereby attenuating vascular inflammation and AAD progression [28]. This indicates that BTK plays a significant role in the inflammatory processes that contribute to aneurysm formation and rupture.

Furthermore, the downregulation of endothelial MerTK (MER proto-oncogene tyrosine kinase) in human aortic aneurysms and dissections (AAAD) has been associated with decreased endothelial cell function and smooth muscle cell phenotypic alterations, promoting aneurysm development [29]. This highlights the importance of maintaining proper tyrosine kinase signaling to prevent pathological changes leading to aneurysm rupture.

Dysregulation of Eph/ephrin signaling has been implicated in various vascular diseases, including AAA. The Ephrin receptor tyrosine kinases, particularly EphB and their ligands ephrin-B2, play significant roles in vascular development and pathology. Ephrin-B2 is crucial for endothelial sprouting and proliferation, as well as supporting vascular smooth muscle cells (VSMCs) [30].

The downregulation of Ephrin receptor tyrosine kinases, particularly ephrin-B2, impacts the progression and potential rupture of abdominal aortic aneurysms (AAA) through several mechanisms. Vascular Smooth Muscle Cell (VSMC) dysfunction. Endothelial cell dysfunction, inflammation, and angiogenesis.

Ephrin-B2 is crucial for the regulation of platelet-derived growth factor receptor β (PDGFRβ) in VSMCs. Downregulation of ephrin-B2 leads to enhanced PDGFRβ internalization and excessive activation of downstream signaling pathways such as MAP kinase and JNK while impairing Tiam1-Rac1 signaling. This results in defective VSMC proliferation and vessel wall integrity, contributing to aneurysm formation and progression [30]. Ephrin-B2 signaling is essential for endothelial cell function, including sprouting, proliferation, and maintaining junctional integrity. Disruption of ephrin-B2 signaling can lead to impaired endothelial cell behavior, contributing to vascular instability and aneurysm development [31,32].

Dysregulation of ephrin-B2 impacts the inflammatory response and angiogenesis, both of which are critical in the pathogenesis of AAA. Altered ephrin-B2 signaling can lead to increased endothelial permeability and pro-inflammatory differentiation, exacerbating vascular inflammation and promoting aneurysm growth [33,34].

In summary, the downregulation of ephrin-B2 disrupts critical signaling pathways in VSMCs and endothelial cells, leading to vascular wall defects, increased inflammation, and impaired angiogenesis, all of which contribute to the progression and potential rupture of AAAs.

The assessment of abdominal aortic aneurysm (AAA) rupture risk traditionally relies on monitoring the maximum diameter of the aneurysm over time, as this has been a primary indicator of potential rupture [35]. However, recent advancements have expanded this approach to incorporate biomechanical modeling and wall stress analysis through computational hemodynamics and solid mechanics simulations, aiming to predict aneurysm-specific wall motion more reliably [36].

Moreover, the integration of novel biomarkers reflecting underlying pathological processes holds promise for enhancing the prediction of rupture risk. Identifying key molecular players involved in aneurysm development and rupture is crucial for improving risk stratification and developing targeted therapies. This approach moves beyond simple diameter-based assessments and aims to capture the complex molecular mechanisms driving aneurysm progression and the weakening of the aortic wall [26].

Although maximum diameter remains a critical factor in assessing AAA rupture risk, incorporating biomechanical modeling and novel biomarkers offers a more comprehensive and accurate prediction of rupture risk.

Urinary biomarkers may serve as early indicators of AAA rupture, and our proteomics discovery might improve disease understanding and risk stratification of AAA. Urine offers a noninvasive and safe source for detecting such biomarkers. Further research would be needed to elucidate any direct connections.

It is important to note that a few limitations apply to the present study. A limited number of patients were included. For this study, we used well-characterized patient samples; however, a key caveat is that only end-stage disease can be assessed, and the inherent variability among patient specimens may affect the findings. Another factor restricting the comprehensive study of the protein repertoire is the current technical constraints. MS/MS and MIB technologies have revolutionized protein analysis and significantly accelerated the process of proteome analysis while maintaining high accuracy and reproducibility. However, the technical complexity of these methods still limits high-throughput capabilities, and lack of sensitivity are aspects that have been restrictive to date. As with omics studies, another issue is that proteins do not act on their own usually but exhibit their activity together with other proteins or ligands, and for the current study, we have focused on the expression levels of the proteins and to further establish the differentially expressed proteins’ putative role in the pathogenesis of RAAA, it is of outermost importance to further investigate those with functional studies. This study is a hypothesis-generating study and aims to identify proteins in the patient’s urine that may have a function in the pathogenesis of AAA rupture, and future studies in larger and independent cohorts will be needed to validate our findings.

## 5. Conclusions

This pilot study presents a promising breakthrough in the diagnosis and surveillance of AAA. We identified six dysregulated protein kinases in the urine proteome of patients with RAAAs, suggesting their potential as urinary biomarkers for early detection of AAA at high risk of rupture. However, these preliminary findings require confirmation in larger prospective cohorts to validate their diagnostic utility and generalizability.

## Figures and Tables

**Table 1:** Patient demographics.

		Non-Ruptured AAA	Ruptured AAA	Total ^1^
Number of patients		5	5	10
Gender: males/females		2/3	2/3	4/6
Age (years)	Median	71.6 (68–75)	75.4 (68–83)	73.5 (68–83)
	(Range)			
Aneurysm diameter (mm)	Median	56.2 (50–72)	57 (49–90)	56.6 (50–90)
	(Range)			
Body mass index		28.2	29.1	28.5
Smoking status (smokers/nonsmokers)		3/2	3/2	6/4
NSAID (Yes/No)		4/1	2/3	6/4
Statins (Yes/No)		5/0	5/0	10/0
Outcome (Alive/Death)		5/0	4/1	9/1

Patient demographics of the two study groups. ^1^ *p*-value was non-significant (*p* > 0.05) for all parameters. Abdominal aortic aneurysm (AAA); nonsteroidal anti-inflammatory drugs (NSAID).

**Table 2:** LFQ Intensity of significant protein kinases.

Gene	Family	Ruptured AAA FFQ Mean (Range)	Non-Ruptured AAA LFQ Mean (Range)	*p*-Value	Majority Protein IDs
*EPHB6* ^1^	TK	21.73	26.44	0.0001	O15197
		(20.99–22.47)	(25.42–27.46)		
*AXL* ^2^	TK	22.73	31.1	0.0001	P30530
		(21.88–23.58)	(29.33–32.87)		
*EPHB4* ^3^	TK	24.16	28.84	0.018	P54760
		(23.31–25.02)	(28.55–29.14)		
*DDR1* ^4^	TK	24.16	27.73	0.031	Q08345
		(22.35–25.98)	(27.11–28.35)		
*EPHA2* ^5^	TK	23.53	28.02	0.013	P29317
		(21.67–25.39)	(26.99–29.06)		
*EPHB3* ^6^	TK	22.81	26.73	0.040	P54753
		(21.87–23.76)	(26.14–27.32)		

^1^ Ephrin type-B receptor 6; ^2^ Tyrosine-protein kinase receptor UFO; ^3^ Ephrin type-B receptor 4; ^4^ Epithelial discoidin domain-containing receptor 1; ^5^ Ephrin type-A receptor 2; ^6^ Ephrin type-B receptor 3. Abdominal aortic aneurysm (AAA); Label-free quantification (LFQ); Tyrosine kinase (TK).

**Table 3:** LFQ Intensity of near significant protein kinases.

Gene	Family	Ruptured AAA FFQ Mean (Range)	Non-Ruptured AAA LFQ Mean (Range)	*p*-Value	Majority Protein IDs
*NME1* ^1^	Metabolic	23.02	25.37	0.055	P15531
		(22.69–23.36)	(24.64–26.11)		
*MAP2K1* ^2^	STE	24.24	25.99	0.060	Q02750
		(23.48–25.01	(25.56–26.43)		
*CSNK1A1* ^3^	CK1	21.90	24.01	0.066	P48729
		(21.51–22.30)	(22.45–25.57)		
*LYN* ^4^	TK	22.04	24.54	0.071	P07948
		(21.75–22.34)	(22.84–26.25)		
*ILK* ^5^	TKL	22.31	23.87	0.082	Q13418
		(21.72–22.90)	(22.52–25.22)		
*CDC42BPB* ^6^	AGC	22.82	25.87	0.086	Q9Y5S2
		(22.44–23.20)	(25.48–26.27)		
*SLK* ^7^	STE	26.79	29.46	0.059	Q9H2G2
		(25.72–27.86)	(28.59–30.34)		

^1^ Nucleoside diphosphate kinase A; ^2^ Dual specificity mitogen-activated protein kinase 1; ^3^ Casein kinase I isoform alpha; ^4^ Tyrosine-protein kinase Lyn; ^5^ Integrin-linked protein kinase; ^6^ Serine/threonine-protein kinase MRCK beta; ^7^ STE20-like serine/threonine-protein kinase. Abdominal aortic aneurysm (AAA); Label-free quantification (LFQ); Tyrosine kinase (TK).

## Data Availability

The original contributions presented in this study are included in the article. Further inquiries can be directed to the corresponding author.

## Abbreviations

The following abbreviations are used in this manuscript:

AAA	Abdominal aortic aneurysms
RAAA	Ruptured abdominal aortic aneurysm
MIBs	Multiplexed kinase inhibitor-conjugated beads
MIB/MS	Quantitative mass spectrometry
NSAID	nonsteroidal anti-inflammatory drugs
LFQ	label-free quantification
TK	Tyrosine kinase
NME1	Nucleoside diphosphate kinase A
MAP2K1	Dual specificity mitogen-activated protein kinase 1
CSNK1A1	Casein kinase I isoform alpha
LYN	Tyrosine-protein kinase Lyn
ILK	Integrin-linked protein kinase
CDC42BPB	Serine/threonine-protein kinase MRCK beta
SLK	STE20-like serine/threonine-protein kinase
EPHB6	Ephrin type-B receptor 6
AXL	Tyrosine-protein kinase receptor UFO
EPHB4	Ephrin type-B receptor 4
DDR1	Epithelial discoidin domain-containing receptor 1
EPHA2	Ephrin type-A receptor 2
EPHB3	Ephrin type-B receptor 3
LYN	Tyrosine-protein kinase Lyn
VSMC	vascular smooth muscle cell
AAAD	Aortic aneurysms and dissections
PDGFRβ	platelet-derived growth factor receptor β
MerTK	MER proto-oncogene tyrosine kinase
